# Ovarian tissue quality assessment and fertility preservation strategies enabled by multi-omics and artificial intelligence: current applications and clinical perspectives

**DOI:** 10.3389/fphys.2026.1789252

**Published:** 2026-03-27

**Authors:** Ruihong Zhang, Hang Du, Tingting Bai, Jie Wang, Yanbin Shi, Xiaoguang Shao, Zhen Huang, Jie Zhang

**Affiliations:** 1 Dalian Medical University, Dalian, Liaoning, China; 2 Zhongshan Clinical Medicine, Dalian University, Dalian, Liaoning, China; 3 Center for Obstetrics, Gynecology and Maternal-Child Reproductive Genetics, Zhongshan Hospital Affiliated to Dalian University, Dalian, Liaoning, China; 4 Dalian Women and Children’s Medical Center (Group), Dalian, Liaoning, China

**Keywords:** artificial intelligence, fertility preservation, multi-omics, ovarian tissue cryopreservation, single-cell analysis

## Abstract

Ovarian tissue cryopreservation (OTC) is essential for fertility preservation in cancer survivors, prepubertal girls, and individuals at high risk of premature ovarian insufficiency (POI). Yet conventional evaluation methods, such as histology and follicle counting, provide limited insight into tissue viability, microenvironmental integrity, molecular injury, and oncologic safety. Recent advances in multi-omics, including transcriptomics, single-cell sequencing, proteomics, and spatial transcriptomics, enable high-resolution characterization of follicular heterogeneity, stromal status, and potential malignant contamination. Concurrently, artificial intelligence (AI) offers automated follicle detection, quantitative tissue assessment, and multimodal prediction models that can support individualized clinical decisions. This review summarizes emerging applications of multi-omics and AI in ovarian tissue quality assessment and highlights their potential to transform fertility preservation strategies. Integrating molecular profiling with AI-based prediction may establish a more precise and intelligent framework for tissue selection, transplantation planning, and reproductive outcome prediction.

## Introduction

The decline of ovarian function and the resulting loss of fertility have become increasingly significant clinical and public health challenges, particularly among cancer survivors, women at high risk of premature ovarian insufficiency (POI), and individuals with hereditary disorders (Kim et al., 2021; Feldberg and Purandare, 2025). The global rise in cancer survivorship, driven by advances in early diagnosis, precision oncology, and multimodal treatments, has markedly expanded the number of young female patients exposed to gonadotoxic chemotherapy or radiotherapy (Angeles Vázquez López, 2024). Over the past two decades, cancer outcomes have improved to such an extent that more than 80% of children and 70% to 90% of adolescents and young adults now survive their malignancy, depending on cancer type and geographic region (Yasmin et al., 2021; Yang et al., 2024). These life-saving treatments, while essential for long-term survival, exert profound and often irreversible damage on primordial follicles and the ovarian stromal microenvironment, resulting in diminished ovarian reserve, endocrine dysfunction, and compromised reproductive potential (Roof et al., 2024; Aboutalebi et al., 2025). Similarly, patients harboring hereditary cancer syndromes such as BRCA1/2 mutations or individuals requiring prophylactic oophorectomy face the threat of POI long before family planning is complete. For children and adolescents, who represent a rapidly growing subgroup of oncology patients, the challenge is even more critical (C et al., 2023; Gootzen et al., 2024). Because they lack mature oocytes and cannot undergo controlled ovarian stimulation, ovarian tissue cryopreservation (OTC) followed by transplantation remains the only feasible approach to preserve future fertility. Although accumulated clinical experience demonstrates that transplanted ovarian tissue can restore endocrine function and has resulted in more than 200 births globally, outcomes remain unpredictable (Najafi et al., 2023). Variability in tissue quality, patient age, underlying disease, and treatment exposures contributes to significant heterogeneity, creating substantial uncertainty for clinicians and patients when planning fertility preservation.

Despite the increasing adoption of OTC worldwide, existing methods for evaluating tissue quality before and after freezing remain rudimentary and often inadequate. Current assessments rely heavily on gross morphological inspection, traditional histology, and basic endocrine markers such as anti-Müllerian hormone (AMH) or follicle-stimulating hormone (FSH). These methods are inherently limited by subjectivity, inter-observer variability, and their inability to capture the complex molecular and functional landscape of ovarian tissue (Spears et al., 2019). Importantly, they inadequately reflect the true biological heterogeneity induced by chemotherapy or radiation. Treatment-related injuries such as DNA double-strand breaks, oxidative stress, microvascular rarefaction, stromal fibrosis, and immune dysregulation frequently remain invisible to routine histopathology (Puy et al., 2023). For cancer survivors, one of the most pressing unmet needs is the detection of minimal residual malignant cells within ovarian tissue, as the potential reintroduction of malignant clones during transplantation poses a significant risk. Current pathological techniques, including immunohistochemistry and flow cytometry, lack the sensitivity required to identify rare malignant cells, particularly in hematologic malignancies (Asadi-Azarbaijani et al., 2016). Consequently, without reliable indicators to assess functional potential, reproductive success, or transplantation safety, clinicians often rely on experience-based judgment, increasing emotional and medical uncertainty for patients who must make irreversible decisions under time pressure.

In recent years, rapid progress in multi-omics technologies has opened new opportunities for exploring ovarian biology with an unprecedented level of detail. High-resolution transcriptomic techniques, including bulk RNA sequencing, single-cell RNA sequencing, and emerging spatial transcriptomics, enable precise mapping of follicular, stromal, immune, and vascular cell populations. These approaches reveal transcriptional states associated with follicle quiescence, activation, atresia, and survival, as well as stromal remodeling and vascular alterations that occur in response to injury (Zhang et al., 2019; Yeh et al., 2024). Proteomic and metabolomic profiling provide complementary insights into oxidative stress pathways, mitochondrial dysfunction, fibrosis-related protein networks, extracellular matrix turnover, and broader metabolic reprogramming that influence tissue viability after cryopreservation and transplantation. Increasing evidence also underscores the significance of epigenetic regulation, including DNA methylation, chromatin accessibility, and histone modifications, in determining follicle fate and sustaining ovarian reserve (Chiang et al., 2020; Du et al., 2020; Li et al., 2021). Together, these molecular layers create a multidimensional landscape that reflects the true cellular and biochemical condition of ovarian tissue and offers substantially greater diagnostic and prognostic power than conventional assessment methods.

In parallel, AI has emerged as a transformative force in reproductive medicine. Deep-learning models enable automated identification of follicles, quantification of stromal fibrosis, and evaluation of vascular architecture with a level of precision that surpasses human visual assessment. When applied to ultrasound or MRI, AI can extract quantitative imaging biomarkers such as ovarian texture, perfusion gradients, and stromal heterogeneity that correlate with functional recovery after transplantation. AI also excels at integrating complex datasets, including multi-omics profiles, digital pathology, imaging features, and longitudinal clinical outcomes. Such predictive modeling can estimate the expected duration of endocrine recovery, the likelihood of achieving pregnancy or live birth, and the safety profile of transplantation for oncology patients (Arlova et al., 2025). This evolution shifts ovarian tissue evaluation from experience-based judgment to a data-driven and individualized framework. The value of AI becomes particularly evident in high-risk settings where sample sizes are small, tissue availability is limited, and clinical decision-making carries significant consequences (Zhang et al., 2025).

Given this evolving landscape, the present review aims to provide a comprehensive synthesis of recent advances in multi-omics analysis and AI-based modeling applied to the assessment of ovarian tissue quality and fertility preservation strategies. We focus specifically on high-risk populations, where robust and precise evaluation is essential to balance reproductive benefit against safety concerns. First, we summarize current clinical practices and highlight persistent limitations in existing assessment frameworks. We then discuss how multi-omics technologies contribute to the development of an emerging “ovarian tissue quality atlas,” providing molecular insight into follicle reserve, treatment-related injury, and potential malignant contamination. Next, we examine how AI can enhance this framework through quantitative pathology, imaging analytics, and integrative predictive modeling to support more consistent and reliable clinical decisions. Finally, we propose a conceptual model integrating an “Ovarian Tissue Quality Score (OTQS)” with personalized strategy recommendations and outline potential pathways toward clinical translation. Together, these developments may facilitate a paradigm shift toward more accurate, safer, and truly individualized fertility preservation for women facing threats to their reproductive future.

## Literature search strategy

To identify foundational literature on ovarian tissue cryopreservation and the rapid progress in multi-omics and artificial intelligence, we searched PubMed and Web of Science for articles published between 2010 and 2025. This time frame was chosen because it represents the period where most clinically relevant applications of single-cell analysis, multi-omics integration, digital pathology, and AI in reproductive medicine emerged. The keywords used included ‘ovarian tissue cryopreservation,’ ‘ovarian tissue transplantation,’ ‘fertility preservation,’ ‘multi-omics,’ ‘transcriptomics,’ ‘single-cell sequencing,’ ‘proteomics,’ ‘metabolomics,’ ‘artificial intelligence,’ ‘digital pathology,’ and ‘radiomics.’ We also screened the reference lists of key reviews and original studies to identify additional relevant work. Older landmark studies were included when they were necessary to understand the development of OTC, but most of the evidence in this review was from recent publications, ensuring that the review reflects current and clinically reliable knowledge.

## Clinical practice of ovarian tissue fertility preservation and the challenges of assessment in high-risk populations

### Indications and overall workflow of ovarian tissue fertility preservation

OTC has become an increasingly important option for women facing fertility threats due to medical conditions or upcoming treatments. The primary indications include patients planning to undergo gonadotoxic chemotherapy or radiotherapy, patients with hematological diseases requiring stem cell transplantation, women at risk of premature ovarian failure (POI), and carriers of genetic cancer syndromes who may require prophylactic oophorectomy (Preservation E.G.G.o.F.F. et al., 2020; Sun et al., 2026). Additionally, for prepubertal girls lacking mature oocytes and adolescents unable to receive hormonal stimulation, OTC remains the only feasible strategy for fertility preservation (Diaz et al., 2022). The overall workflow of OTC typically begins with the assessment of ovarian reserve, medical risks, and treatment urgency, followed by the laparoscopic operation for obtaining ovarian cortical tissue. The obtained tissue fragments are cryopreserved using either slow freezing or vitrification methods, both of which aim to minimize ice crystal damage and maintain follicular activity (Wang Y. et al., 2024). When patients later hope to restore ovarian function or fertility, the frozen tissue will be thawed for *in situ* transplantation (such as in the pelvic cavity) or ectopic transplantation (such as subcutaneously). The subsequent monitoring focuses on the recovery of endocrine function, follicular development, and the possibility of natural or assisted conception (Finkelstein et al., 2025). Despite a well-established operational framework, significant differences in efficacy exist among individual patients, highlighting the importance of implementing strict and individualized quality assessment.

Several ovarian tissue-specific clinical studies have reported benchmarks for fertility preservation outcomes and linked evaluation methods to real reproductive results. Most published cohorts have used slow-freezing techniques, which remain the main method in clinical practice, with vitrification becoming increasingly reported but with fewer large outcome series. A recent systematic review of orthotopic ovarian tissue transplantations found that all 92 participants had tissue cryopreserved by slow freezing, with pregnancy rates of 81.3%, 45.5%, and 66.7% and corresponding live birth rates of 56.3%, 18.2%, and 66.7% among different tissue size groups, and median restoration of endocrine function within 3–4 months after grafting (Diaz et al., 2022). A meta-analysis showed that mean pregnancy and live birth rates following ovarian tissue transplantation were approximately 44% and 35%, respectively (Wang Y. et al., 2024). In a large cohort from Victoria, Australia (1995–2022), 48 of 593 women underwent ovarian tissue transplantation, and overall pregnancy outcomes were similar between transplanted and non-transplanted groups, with 192 neonates reported from 114 women, highlighting the long-term feasibility of OTC for fertility preservation (Finkelstein et al., 2025). These clinical outcomes have been assessed mainly using classical evaluations, though more recent studies are beginning to explore molecular and imaging biomarkers with traditional histology to better stratify tissue quality before and after cryopreservation. Taken together, these reports emphasize that OTC can restore ovarian function and lead to live births in a meaningful fraction of cases, but variability in patient population, technique, and follow-up underscores the need for standardized multi-omics and AI-enhanced assessment strategies that link biological signatures to clinical success.

## Limitations of current tissue quality assessment approaches

Despite growing clinical demand, current methods used to assess the quality of ovarian tissue before and after cryopreservation remain limited in scope and precision. Structural evaluation—such as counting primordial follicles, observing cortical thickness, grading stromal fibrosis, or describing vascular density—still depends largely on manual interpretation of histological slides (Goldenberg et al., 2025). This inherently subjective approach introduces variability between observers and clinical centers, as differences in sectioning, staining, and interpretation can lead to inconsistent assessments. As a result, traditional structural evaluation offers only a limited and sometimes misleading view of true tissue viability. Importantly, morphology alone cannot capture early molecular or microenvironmental disturbances—such as oxidative stress, vascular impairment, or subtle stromal changes—that influence graft performance after transplantation (Guo et al., 2024). Therefore, structural markers are often poor predictors of whether ovarian tissue will successfully regain endocrine function or how long such recovery can be maintained.

Functional assessment presents additional challenges. Common indicators such as serum FSH, AMH, and antral follicle counts fluctuate substantially and may not reliably reflect the biological integrity of cryopreserved tissue. Imaging modalities like ultrasound or MRI can reveal ovarian size and perfusion but lack the sensitivity needed to detect microstructural or molecular damage induced by chemotherapy or radiation (Zhao et al., 2023). Safety evaluation is even more complex, especially for cancer survivors. Detecting minimal residual malignant cells within ovarian tissue is extremely difficult with standard pathology or immunohistochemistry, and false-negative results may place patients at risk of recurrence after transplantation (Andersen et al., 2012; Zver et al., 2022).

Moreover, evidence from hematologic oncology highlights how limited traditional methods are in detecting minimal residual malignant cells. For instance, routine pathology and immunohistochemistry can be used to generally detect malignant cells only when they account for at least 1–5% of a specimen, and standard multiparameter flow cytometry usually reaches a sensitivity of about 10–^3^ to 10^-4^ (Borowitz et al., 2008; Campana, 2010). However, next-generation sequencing-based MRD assays can be used to improve this and typically achieve detection limits in the range of 10–^5^ to 10^-6^ (Wood et al., 2018; Song et al., 2025). Single-cell sequencing represents another important facet, with studies in acute lymphoblastic leukemia and acute myeloid leukemia showing that scRNA-seq can reveal rare leukemic subpopulations, including therapy-resistant or relapse-associated clones, that are not identifiable by morphology, IHC, or flow cytometry (Van Galen et al., 2019; Wu et al., 2020). These findings demonstrate that single-cell technologies provide approximately one to two orders of magnitude higher sensitivity than routine histopathology. Thus, incorporating such approaches into ovarian tissue evaluation could therefore improve the detection of malignant contamination, particularly for patients with leukemia, and help reduce the risk of reintroducing residual disease during autotransplantation.

Collectively, these limitations highlight the inadequacy of conventional assessments in capturing the true biological state of ovarian tissue. They also explain why many patients experience uncertain or inconsistent outcomes despite undergoing the same procedural steps.

## The unmet need for data-driven, predictive, and interpretable assessment in high-risk patients

For high-risk populations, the central challenge in ovarian tissue preservation extends well beyond determining whether the procedure is technically feasible. Clinicians must discern which patients are likely to benefit, which may face substantial risks, and how to optimize both the timing and procedural aspects of tissue retrieval, cryopreservation, and transplantation. As treatment exposures, disease backgrounds, and clinical trajectories vary widely among patients, decision-making increasingly requires assessment systems that are quantitative, reproducible, and capable of predicting outcomes such as early graft function, long-term endocrine recovery, and reproductive potential (Lee et al., 2021; Li Y. et al., 2024; Emrich et al., 2025).

A robust evaluation framework must therefore clarify whether a given tissue fragment retains sufficient biological value to justify preservation, whether autotransplantation is advisable in the context of prior treatments, how much tissue should be transplanted at a given time to maximize functional recovery, and whether the sample carries any risk of malignant contamination. Addressing these questions is essential for moving fertility preservation from an experience-driven approach to one grounded in evidence and precision (Ruan et al., 2024).

**Figure 1:**
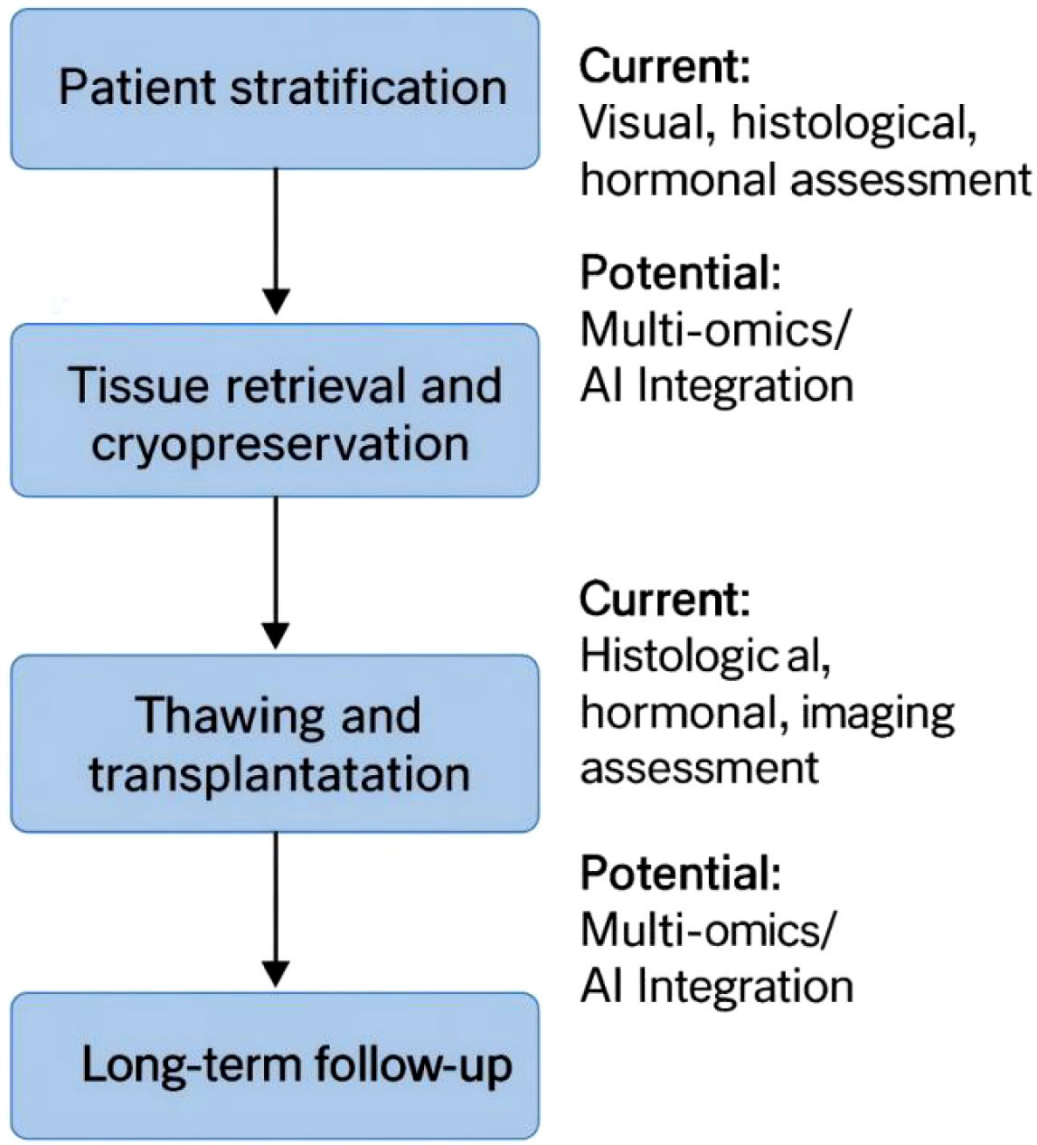
Key steps of the ovarian tissue cryopreservation workflow and current versus potential assessment approaches. The figure outlines the main steps of ovarian tissue cryopreservation (patient stratification, tissue retrieval and cryopreservation, thawing and transplantation, and long-term follow-up). For each step, the figure contrasts current clinical practice, primarily visual inspection, histological assessment, hormonal markers, and imaging, with potential future approaches incorporating multi-omics profiling and AI-based analysis.

Traditional morphological and hormonal indicators are unable to detect the patterns of tissue damage, molecular disorders, and changes in the microenvironment in high-risk patients (Van Galen et al., 2019; Wu et al., 2020). In contrast, multi-omics technologies can provide molecular characteristics related to follicle survival ability, matrix integrity, and damage induced by cryopreservation, providing a more objective basis for evaluating tissue suitability. Therefore, by combining multi-omics data that can reveal the deep molecular features of the tissue with AI models that are proficient in integrating analysis and prediction, a highly promising new paradigm is provided for achieving personalized decisions in key aspects of tissue acquisition, preservation, and transplantation (Li Y. et al., 2024; Emrich et al., 2025). These innovative technologies collectively constitute the key decision-making nodes in the ovarian tissue preservation pathway (**Figure 1**), and effectively fill the important gaps in current individualized clinical decision-making for high-risk populations.

## Multi-omics construction of an “Ovarian Tissue Quality Atlas”: evidence from molecular profiling

### Major multi-omics dimensions applicable to ovarian tissue biology

Recent advances in multi-omics have provided a more detailed understanding of ovarian tissue quality than what can be achieved with morphology alone. Modern studies now characterize ovarian tissue across several molecular layers, such as transcriptomic, proteomic, metabolomic, and epigenomic, with each contributing important information relevant to follicle viability, stromal integrity, and the effects of prior gonadotoxic treatment.

Transcriptomic profiling, including both bulk and single-cell RNA sequencing, has clarified the cellular composition and functional states of the ovarian cortex (Zhang et al., 2024; Zhu et al., 2025). These methods can help distinguish granulosa cells, theca cells, stromal fibroblasts, endothelial cells, and multiple immune populations, and they reveal transcriptional programs linked with follicle activation, atresia, or chemotherapy-related injury. Single-cell approaches have also been applied in cancer-treatment contexts to identify early molecular disturbances that precede visible structural damage, offering a clinically relevant means of detecting subtle changes before and after cryopreservation (Li X. et al., 2024).

Proteomic analyses also provide complementary information as they can be used to identify changes in structural proteins, cytoskeletal components, mitochondrial enzymes, and mediators of stress or inflammation. Several recent studies examining ovarian tissue collected before and after chemotherapy have reported alterations in extracellular matrix proteins, oxidative-stress pathways, and vascular-related proteins, which are features that correlate with diminished follicle survival after transplantation (Grosbois et al., 2025). These protein-level changes often remain invisible on routine histology, supporting the clinical value of proteomic assessment in high-risk patients.

Metabolomic profiling can be used to identify shifts in energy metabolism, lipid handling, redox balance, and mitochondrial function, all of which are relevant to the resilience of ovarian tissue following cryopreservation (Chen et al., 2022). Studies analyzing ovarian tissue or associated media have shown that chemotherapy and ischemia-reperfusion injury can disrupt glucose utilization, amino-acid signatures, and antioxidant metabolites (Spears et al., 2019). These biochemical patterns may also help identify tissue that is less likely to regain endocrine function or support follicle development after transplantation.

Although used less frequently, epigenomic analyses have provided important insights into the regulation of follicle quiescence, activation, and long-term oocyte competence (Li, 2021). Reports examining DNA-methylation changes and chromatin accessibility in ovarian tissue have identified age-related and treatment-related epigenetic alterations that influence follicle behavior (Chen et al., 2024). These findings suggest that epigenomic markers could serve as early indicators of tissue decline or maladaptive activation, with potential value in pre-transplant quality assessment.

Together, these multi-omics layers outline a structured and clinically relevant framework that expands beyond classical histology and provides a more comprehensive basis for evaluating ovarian tissue health.

## Core “quality dimensions” revealed by multi-omics approaches

The foundational determinant of ovarian tissue quality is the size and integrity of the primordial follicle pool. Multi-omics studies have elucidated transcriptional programs and molecular pathways that govern primordial follicle maintenance, activation thresholds, and survival mechanisms (Lim et al., 2024). Single-cell data can map follicle developmental trajectories, identify subpopulations with higher reproductive potential, and detect molecular signatures associated with oocyte competence (Ziegenhain et al., 2017). These attributes provide objective criteria for determining whether tissue is biologically “worth preserving,” particularly in high-risk patients whose follicle reserve may be severely compromised.

For cancer survivors and other high-risk groups, assessing the extent of therapy-related damage is essential. Chemotherapy and radiotherapy induce a spectrum of molecular injuries—including DNA double-strand breaks, oxidative stress responses, mitochondrial dysfunction, and stromal inflammation—that may not be apparent histologically. Proteomic and transcriptomic analyses allow quantitative evaluation of these injury signatures, such as fibrosis-related pathways, pro-apoptotic markers, or inflammatory cytokine networks. These indicators help estimate the residual functional capacity of the tissue and predict the likelihood of endocrine recovery or sustained follicular activity after transplantation (Hwang et al., 2018; Zhang et al., 2020).

The ovarian cortex is a complex microenvironment consisting of immune cells, vascular networks, extracellular matrix components, and support cells that regulate follicular survival. Spatial transcriptomics and high-resolution single-cell approaches can reveal microenvironmental disruptions, such as aberrant immune infiltration, vascular rarefaction, or fibrotic remodeling, all of which impair tissue viability. Importantly, in oncology patients, these technologies offer unprecedented power to detect rare malignant clones, characterize their transcriptional profiles, and distinguish them from benign cell populations (McGinnis et al., 2019). This molecular stratification is critical for defining transplantation safety and minimizing the risk of reintroducing malignant disease.

Collectively, these three quality dimensions-follicle reserve, injury burden, and microenvironmental integrity-form the biological foundation of a systematic assessment framework.

## From multi-omics data to an “Ovary Quality Atlas”

**Figure 2:**
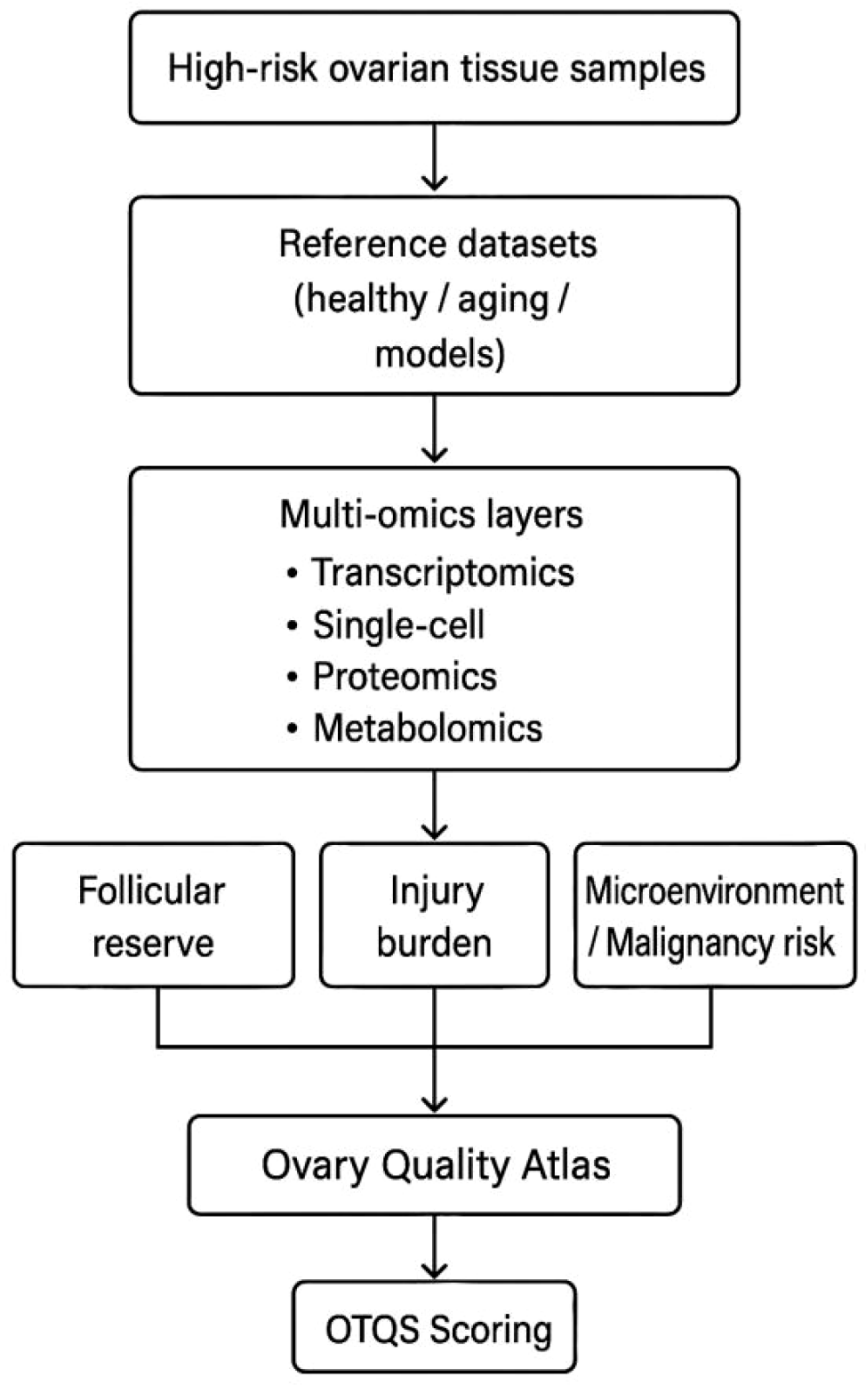
Construction of the ovarian tissue quality atlas and derivation of the Ovarian Tissue Quality Score (OTQS). This figure illustrates the workflow for building an ovarian tissue quality atlas. High-risk ovarian tissue samples are compared against reference datasets derived from healthy, aging, or disease-model tissues. Multi-omics layers, including transcriptomics, single-cell sequencing, proteomics, and metabolomics, are integrated to evaluate three major dimensions of tissue status: follicular reserve, injury burden, and microenvironmental or malignancy-related risk. These components form the basis of the ovarian tissue quality atlas, from which OTQS can be generated.

As illustrated in **Figure 2**, integrating transcriptomic, proteomic, metabolomic, and epigenomic data enables the construction of a comprehensive “Ovary Quality Atlas,” a multi-layered representation of ovarian tissue health. This framework maps the molecular landscape of ovarian tissue by capturing key dimensions, including follicular reserve, functional potential, stromal and vascular integrity, and treatment-related injury signatures (Fonseca et al., 2023). By incorporating reference datasets derived from healthy reproductive-age individuals, aging populations, and disease models, the atlas situates high-risk patients within a broader biological continuum, allowing their molecular profiles to be interpreted in a clinically meaningful context.

Beyond descriptive value, the atlas serves as a unifying platform for stratifying patients across diverse high-risk groups—such as oncology survivors, individuals with hereditary disorders, and those at elevated risk of POI—by aligning individual tissue signatures with established molecular patterns (Wang et al., 2020). This approach supports more objective and standardized evaluation of tissue viability and transplantation potential. Taken together, these integrated data layers form the conceptual foundation for developing an OTQS, which transforms multidimensional molecular information into a clinically interpretable metric. A validated OTQS could substantially enhance decision-making by helping clinicians determine whether ovarian tissue should be preserved, whether autotransplantation is advisable, and which strategies may optimize reproductive and endocrine outcomes while limiting associated risks.

Conceptually, the integration of multi-omics in OTC should be viewed as the development of a Reference Atlas rather than a single molecular test, as it is built from high-quality ovarian tissues collected across different ages, clinical backgrounds (such as pre-treatment and post-treatment), and processing states (i.e., fresh, cultured, cryopreserved, rewarmed) to allow ovarian tissue being assessed in clear molecular landscape and supports the identification of key signatures linked to tissue quality. These signatures repeatedly correlate with important biological features, including follicular competence, stromal and vascular integrity, immune activation, oxidative or ischemic stress, and DNA-damage responses (He et al., 2024; Zhang et al., 2026). After these signatures are compared with clinical outcomes such as graft function, endocrine recovery, and follicle development, they provide a stable molecular basis for standardizing assessment across centers and guide atlas-based tissue stratification (He et al., 2024).

The Reference Atlas also helps translate molecular findings into indicators that can be detected non-invasively or with minimal tissue handling. In this regard, radiomics and radiogenomics offer one possible route. For instance, studies in oncology show that imaging features, such as texture and heterogeneity, can reflect underlying gene-expression programs and microenvironmental patterns, including hypoxia-related pathways, suggesting that similar “imaging-to-signature” models could be adapted for ovarian tissue evaluation (He et al., 2024; Shao et al., 2025; Zhang et al., 2026). Another practical direction involves metabolite signals from tissue-associated fluids. In organ preservation, biomarkers such as flavin mononucleotide (FMN) have been used to assess mitochondrial injury, with perfusate metabolomics identifying metabolite patterns linked to later graft outcomes (Liu RX. et al., 2023; Rhee, 2023; Sun et al., 2025). Moreover, in reproductive medicine, culture-media metabolomics has long been explored as a way to assess embryo viability and developmental competence, demonstrating that extracellular metabolites can reflect tissue function (Šmak et al., 2025). When these imaging findings and metabolite patterns are matched to atlas-defined molecular signatures, they provide useful surrogate indicators that complement histology, reduce the need for invasive sampling, and allow monitoring of tissue condition during culture, cryostorage, and rewarming. In addition, recent evidence from perfusate metabolomics in organ preservation further strengthens this concept, with a study demonstrating that standardized quantification of FMN can serve as a reliable indicator of mitochondrial injury and tissue viability, illustrating how metabolite-based readouts can be incorporated into practical decision tools in clinical workflows (Dong et al., 2026).

Lastly, recent advances in multi-omics integration and atlas-based modeling further support this direction. Studies using cross-modal data fusion and reference atlas construction in complex biological systems demonstrate how combining transcriptomic, proteomic, and spatial datasets can improve tissue-state annotation and enhance biological interpretability (Tian et al., 2025). These developments provide an important methodological foundation for expanding and refining ovarian tissue quality atlases.

## Artificial intelligence—enabled ovarian tissue assessment and decision-making in fertility preservation

### Advancing quantitative and standardized evaluation through AI in pathology and imaging

Artificial intelligence has emerged as a transformative approach to overcoming the subjectivity and variability that characterize traditional ovarian tissue assessment. In digital pathology, rapid progress in convolutional neural networks (CNNs) and related deep-learning architectures has enabled automated identification of primordial and growing follicles with levels of precision that rival expert histologists (Tal and Seifer, 2017; Wang YL. et al., 2024). Beyond simple follicle detection, advanced models now quantify cortical thickness, characterize stromal fibrosis, assess vascular and extracellular matrix organization, and detect subtle morphological aberrations that may reflect early cryoinjury or chemotherapy-induced damage (Qian et al., 2025). These systems can process thousands of histological images within minutes, allowing for efficient evaluation of entire tissue fragments rather than isolated representative sections, which significantly reduces sampling bias and enhances reproducibility across clinical laboratories (İnik et al., 2019).

Parallel advances in AI-enhanced medical imaging further expand the potential for noninvasive assessment of ovarian tissue. Deep-learning algorithms applied to pelvic ultrasound and magnetic resonance imaging (MRI) can extract quantitative features that describe ovarian volume, vascular perfusion, microstructural texture, and even inferred markers of tissue metabolism (Breen et al., 2023; Wang X. et al., 2025). These imaging-derived phenotypes correlate with key clinical outcomes, including graft revascularization, endocrine recovery, and follicular activation after transplantation. Emerging radiomics approaches integrate high-dimensional imaging features with clinical and laboratory data, enabling the prediction of ovarian reserve decline or post-transplant functional capacity with greater accuracy than conventional imaging interpretation alone (Park et al., 2020; Li et al., 2025).

AI-driven pathology and imaging tools also offer distinct advantages for high-risk populations, where the amount of available ovarian tissue is often limited and precision is essential for safe clinical decision-making. Automated quantification provides standardized metrics that facilitate longitudinal monitoring and allow comparisons between different tissue fragments, time points, and clinical centers. These technologies enhance diagnostic confidence, improve workflow efficiency, and reduce interobserver variability. Although further validation and multi-center harmonization are necessary, AI-supported evaluation is rapidly becoming a cornerstone of data-driven fertility preservation and is poised to play an increasingly central role in guiding tissue selection and transplantation strategies (Segers et al., 2020; Liu et al., 2024).

### Predictive modeling through integration of multi-omics and clinical data

Beyond tissue characterization, AI excels in integrating heterogeneous data to generate predictive models relevant to clinical outcomes. Machine learning approaches—including feature selection algorithms, dimensionality reduction techniques, and ensemble models—can extract critical variables from multi-omics profiles, imaging data, and clinical histories to forecast key reproductive endpoints. Such models may predict the duration of ovarian endocrine recovery following transplantation, the likelihood of achieving pregnancy or live birth, and the probability of sustained follicular activity (Liu X. et al., 2023).

For high-risk patients, AI offers particular value in evaluating transplantation safety. By combining molecular signatures of malignancy with histopathological features and clinical parameters, predictive models may help estimate the risk of reintroducing malignant cells, a concern that is especially prominent for survivors of hematologic cancers. Moreover, AI can assist in pre-cryopreservation decision-making by estimating the balance between potential fertility benefits and treatment-related risks, ultimately guiding clinicians in determining whether tissue preservation is likely to be worthwhile (Park et al., 2023). These predictive tools support more individualized and evidence-based counseling, helping patients and clinicians navigate complex choices.

### Challenges of data scarcity and potential technical solutions

AI applications in ovarian tissue assessment face a fundamental challenge since the amount of available data remains limited. Ovarian tissue samples are small, the number of cryopreserved cases remains modest, and multimodal datasets combining histology, imaging, and molecular profiles are not yet widely available. These constraints can reduce model robustness and limit the generalizability of algorithms across institutions (Khosravi et al., 2024; Koetzier et al., 2024). Several technical strategies used in medical imaging may help address this problem. Transfer learning allows models pretrained on large imaging datasets to adapt to small ovarian tissue datasets, improving feature extraction and reducing overfitting; this paradigm is widely used across digital pathology and gynecologic imaging workflows when training data are constrained (Cossio et al., 2025; Wang J. et al., 2025; Ji et al., 2026). Another potential approach is the use of synthetic data. Generative adversarial networks (GANs) and related generative methods can produce realistic histology or imaging variants that expand training inputs and improve model resilience, particularly when rare injury patterns or specific stromal features are otherwise underrepresented (Khosravi et al., 2024; Koetzier et al., 2024; Ruiz-Casado et al., 2024; Lesmes-Leon et al., 2025). Although these approaches require careful validation, they provide feasible pathways to mitigate data scarcity while maintaining model reliability.

### A conceptual framework for “OTQS + personalized strategy recommendation”

Building on insights from multi-omics profiling and AI integration, a conceptual framework for an OTQS and personalized fertility preservation strategy emerges. In this framework, data inputs include molecular profiles, digital pathology outputs, imaging-derived biomarkers, and patient-specific clinical information. Using validated predictive models, these inputs are synthesized into a quantitative OTQS that reflects overall tissue viability, functional potential, and safety considerations (Moustakli et al., 2025).

The downstream applications of this scoring system are multifold. The OTQS could help clinicians determine whether OTC is advisable for a given patient and whether it should be combined with oocyte or embryo cryopreservation to optimize reproductive outcomes. The score may also inform the extent of tissue retrieval, the choice of cryopreservation technique, and the timing and amount of tissue selected for transplantation (Lobo et al., 2025). For high-risk individuals, the system could additionally provide safety alerts—such as potential malignant contamination—allowing clinicians to weigh the risks of transplantation against non-transplant alternatives. Importantly, AI-driven recommendations are intended to support, rather than replace, clinical judgment, ensuring that decision-making remains anchored in individualized care and multidisciplinary expertise.

### Clinical translation pathways and practical considerations

**Figure 3:**
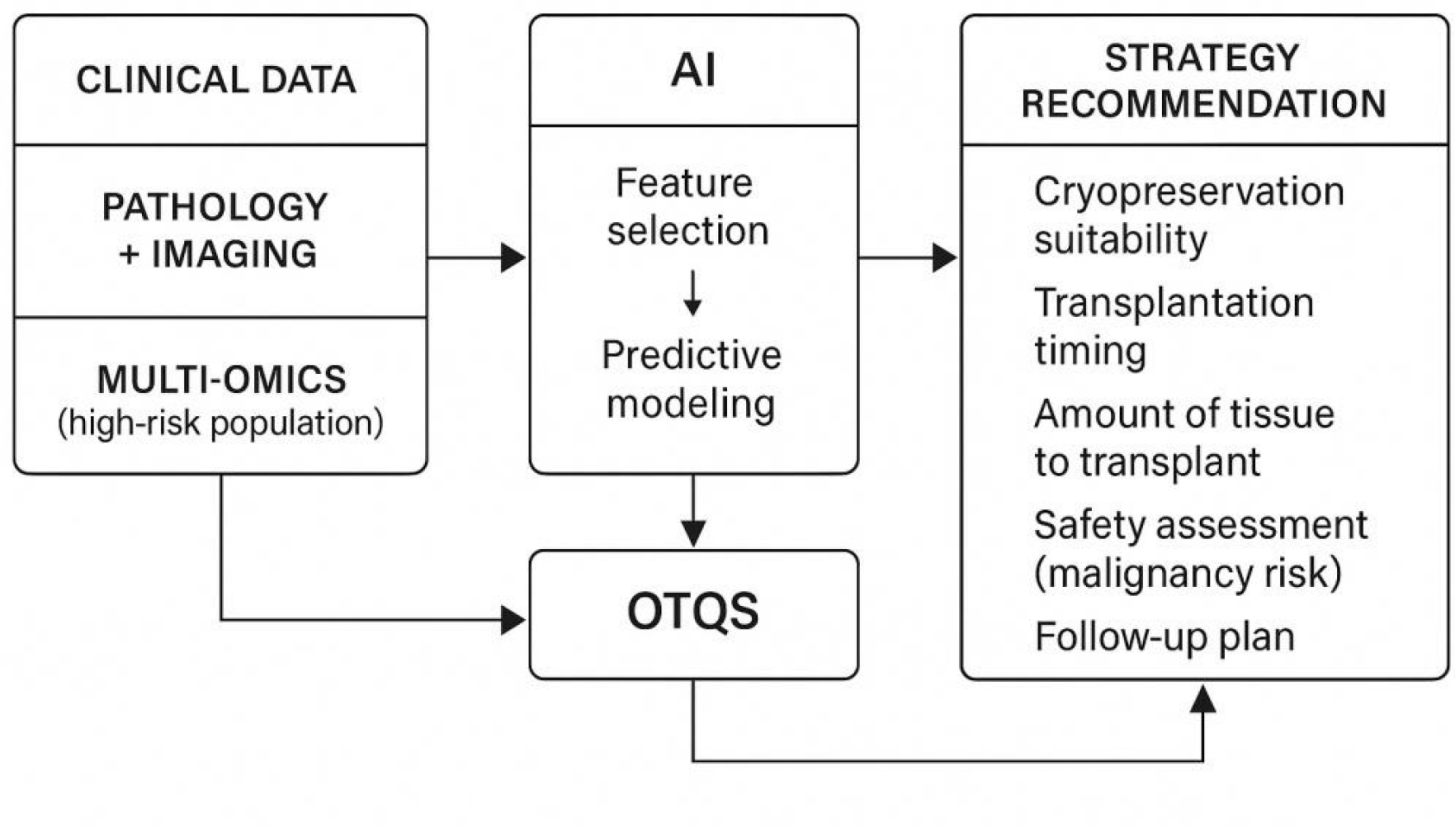
AI-enabled multi-omics framework for ovarian tissue quality assessment and fertility preservation decision-making.

Realizing the full potential of AI-enabled ovarian tissue assessment requires a structured and deliberate pathway toward clinical implementation. As illustrated in **Figure 3**, successful translation begins with the development of prospective, well-annotated, and ethically governed datasets, especially those derived from high-risk patient groups (Milne-Ives et al., 2020). These datasets form the foundation for model training, validation, and external reproducibility. Collaborative, multicenter data sharing is equally important to ensure that AI models remain generalizable and representative of diverse clinical populations.

Progress also depends on strong interdisciplinary coordination. Reproductive medicine specialists, oncologists, pediatricians, pathologists, data scientists, and AI engineers must work together to define clinically meaningful endpoints, standardize data acquisition, optimize algorithm performance, and confirm predictive reliability (Wang et al., 2023; Bedi et al., 2025). Ethical principles such as data privacy, informed consent, transparency of algorithmic processes, and the protection of vulnerable individuals must guide every stage of development and deployment (Topol, 2019). Recent work in AI governance and biomedical data standards indicates that successful translation requires not only algorithmic accuracy but also robust data quality, transparent workflows, and scalable infrastructure across institutions. A recent study emphasized that cross-center reproducibility, harmonized data acquisition, and continuous performance monitoring are essential for reliable clinical deployment of AI-enabled systems (Wang R. et al., 2024). These insights are directly relevant to multi-omics and AI integration in ovarian tissue assessment. As AI-informed recommendations increasingly influence decisions related to fertility preservation, maintaining human oversight and ensuring fair and responsible clinical access will be crucial for safe and effective adoption.

## Conclusion

### Overall value and significance

The integration of multi-omics technologies and artificial intelligence offers an unprecedented opportunity to redefine how ovarian tissue is evaluated for fertility preservation. By bridging macroscopic morphology, molecular mechanisms, and clinical outcomes, these approaches enable a truly comprehensive assessment framework that surpasses the limitations of traditional methods. Multi-omics provides fine-grained insights into follicular health, stromal dynamics, and microenvironmental integrity, while AI introduces quantitative rigor and predictive capability across pathology, imaging, and clinical decision-making. For high-risk populations—such as cancer survivors, patients with genetic conditions, and individuals at elevated risk of POI—this integrated framework is especially valuable. It supports more accurate identification of candidates who are likely to benefit from OTC and transplantation, enhances safety assessment in complex clinical scenarios, and improves the precision of personalized fertility preservation strategies.

### Current limitations and challenges

Despite their considerable promise and the encouraging advances to date, the evidence base for multi-omics and AI in this field remains largely exploratory. Most studies are limited by factors such as small sample sizes, retrospective or single-center designs, and significant methodological heterogeneity. Direct multi-omics analysis of ovarian tissues from high-risk patients is still relatively rare, and artificial intelligence-based prediction models often lack external validation. Differences in specimen processing methods, cryopreservation protocols, sequencing platforms, and computational processes further increase the complexity of comparisons between different studies. Additionally, the implementation of complex data-driven tools faces ethical, regulatory, and logistical challenges. Especially when involving vulnerable groups such as minors, careful consideration is needed, and attention must be paid to data governance, algorithm transparency, and fair access. Overall, these limitations indicate that the transition from proof-of-concept studies to validated, clinically applicable tools is still in its infancy.

### Future directions and translational pathways

Looking ahead, to advance this field toward routine clinical applications, several key steps need to be taken. Firstly, establish a large-scale, multi-center, and forward-looking ovarian tissue biobank with maintenance functions, focusing on high-risk populations, and connecting it with detailed clinical and long-term outcome data. These biobanks will provide large-scale, well-annotated datasets for building and validating comprehensive ovarian quality maps and standardized OTQS. Secondly, establish feasible methodological standards. Develop and adopt uniform procedures for each step from tissue collection and cryopreservation to multi-omics analysis, digital pathology, and artificial intelligence model reporting, to ensure reproducibility, promote data sharing, and facilitate comparisons between different institutions. Thirdly, advance and validate artificial intelligence algorithms. Beyond the prototype development stage, future work must focus on rigorous internal and external validation of artificial intelligence models using independent datasets. Research should aim to improve the interpretability, robustness, and fairness of the models. The ultimate goal is to develop these tools into reliable, clinically trustworthy decision support systems. Ultimately, by systematically addressing these challenges, multi-omics and artificial intelligence technologies will mature and be more seamlessly integrated into reproductive medicine, potentially transforming fertility preservation from an experience-driven practice to a discipline guided by precision. Through achieving more accurate risk assessment, safer and more personalized treatment strategies, and ultimately providing patients with better fertility outcomes and improved quality of life.
